# Intravascular imaging of angioplasty balloon under-expansion during pre-dilation predicts hyperelastic behavior of coronary artery lesions

**DOI:** 10.3389/fbioe.2023.1192797

**Published:** 2023-05-22

**Authors:** Arash Ghorbannia, John F. LaDisa

**Affiliations:** ^1^ Section of Pediatric Cardiology, Department of Pediatrics, Medical College of Wisconsin, Milwaukee, WI, United States; ^2^ Herma Heart Institute, Children’s Wisconsin, Milwaukee, WI, United States; ^3^ Department of Biomedical Engineering, Marquette University and The Medical College of Wisconsin, Milwaukee, WI, United States; ^4^ Department of Physiology, Milwaukee, WI, United States; ^5^ Division of Cardiovascular Medicine, Medical College of Wisconsin, Milwaukee, WI, United States

**Keywords:** percutaneous coronary intervention, material characterization, stent deployment, coronary artery disease, lesion stiffness

## Abstract

**Introduction:** Stent-induced mechanical stimuli cause pathophysiological responses in the coronary artery post-treatment. These stimuli can be minimized through choice of stent, size, and deployment strategy. However, the lack of target lesion material characterization is a barrier to further personalizing treatment. A novel *ex-vivo* angioplasty-based intravascular imaging technique using optical coherence tomography (OCT) was developed to characterize local stiffness of the target lesion.

**Methods:** After proper institutional oversight, atherosclerotic coronary arteries (*n* = 9) were dissected from human donor hearts for *ex vivo* material characterization <48 h post-mortem. Morphology was imaged at the diastolic blood pressure using common intravascular OCT protocols and at subsequent pressures using a specially fabricated perfusion balloon that accommodates the OCT imaging wire. Balloon under-expansion was quantified relative to the nominal balloon size at 8 ATM. Correlation to a constitutive hyperelastic model was empirically investigated (*n* = 13 plaques) using biaxial extension results fit to a mixed Neo-Hookean and Exponential constitutive model.

**Results and discussion:** The average circumferential Cauchy stress was 66.5, 130.2, and 300.4 kPa for regions with <15, 15–30, and >30% balloon under-expansion at a 1.15 stretch ratio. Similarly, the average longitudinal Cauchy stress was 68.1, 172.6, and 412.7 kPa, respectively. Consequently, strong correlation coefficients >0.89 were observed between balloon under-expansion and stress-like constitutive parameters. These parameters allowed for visualization of stiffness and material heterogeneity for a range of atherosclerotic plaques. Balloon under-expansion is a strong predictor of target lesion stiffness. These findings are promising as stent deployment could now be further personalized via target lesion material characterization obtained pre-operatively.

## 1 Introduction

Coronary artery disease (CAD) is the most common form of cardiovascular disease and the leading cause of death in the US (Murphy et al., 2021). CAD develops due to the accumulation of atherosclerotic tissue within the artery wall, and it can take decades before a flow-limiting narrowing that requires intervention presents. With the advent of coronary stents, percutaneous coronary intervention (PCI) is now the standard treatment for many CAD patients. However, poor clinical outcomes are still common in the form of restenosis (Aoki and Tanabe, 2021) and, to a lesser extent, stent thrombosis (Claessen Bimmer E. et al., 2014). These outcomes are particularly prone after treating plaques with adverse material characteristics, such as thin fibrous cap with high rupture vulnerability or highly calcific plaques with generally stiffer structure (Yang et al., 2022).

The widespread availability of medical imaging and computer models has brought structural and hemodynamic simulation to the leading edge of clinical decision making, providing insight into the impact of patient-specific characteristics (Rikhtegar et al., 2014; Chiastra et al., 2016; Ebrahimi and Fallah, 2022). While current imaging modalities are mostly geared toward morphology characterization, knowing material properties of the target lesion is a requirement to study plaque structural stress. Importantly, structural stress correlates strongly with poor outcomes of stent deployment including restenosis, stent thrombosis and plaque rupture (Teng et al., 2014; Kolandaivelu and Rikhtegar, 2016; Costopoulos et al., 2017; Conway et al., 2021; Kadry et al., 2021). Local artery stiffness also predicts delivery balloon under-expansion and elastic recoil through stent-plaque strain energy exchange after balloon deflation (Aziz et al., 2007). Consequently, suboptimal final artery geometry and associated adverse mechanical stimuli, such as wall shear stress (LaDisa et al., 2004; 2005; Nakagawa et al., 2014), can trigger proinflammatory, prothrombotic, and proatherogenic responses that progress to poor clinical outcomes (Thondapu et al., 2017).

An optimal PCI procedure can be defined as recovering coronary flow reserve with minimal stent-induced mechanical stimuli. In this regard, stent selection, deployment strategy, and controlled recoil all become important and are influenced by local morphology and material properties of the target lesion. However, there is a lack of feasible tools for patient-specific hyperelastic material characterization of coronary plaques. Importantly, current state-of-the-art virtual simulations have the potential to significantly improve clinical decision making toward personalized PCI by incorporating high-fidelity morphology and material characteristics of the target lesion. However, recent attempts for image-based characterization of plaque material properties have been limited to *ex vivo* approaches or multimodal intravascular imaging. Related work has also focused on physiological intravascular pressures (Guvenir Torun et al., 2021; Narayanan et al., 2021), while nonlinearity of the plaque deformation (i.e., hyperelastic behavior) is best quantified beyond physiological deformations as applied during PCI. In this paper a novel approach is presented using OCT imaging through angioplasty balloons at pre-dilation to 8 ATM. Pre-dilation inherently provides physical contact with the target lesion, which provides a unique opportunity for patient-specific material characterization within the PCI range of deformations. The feasibility of this method is empirically investigated using an *ex vivo* set up for coronary atherosclerotic human arteries.

## 2 Methods

### 2.1 OCT integrated angioplasty balloon

A specially fabricated 4 × 40 mm angioplasty balloon with 3 Fr lumen was created to accommodate the advancement of a 2.7 Fr OCT catheter (Abbott, Dragonfly™ lineOPTIS™ Imaging Catheter, C408645) and allow for intravascular imaging through the transparent balloon surface during pre-dilation. The fabricated angioplasty balloon was then used in an *ex vivo* set up for intravascular imaging of atherosclerotic coronary arteries obtained from human donor hearts as described below.

### 2.2 Coronary artery harvest and imaging protocol

**FIGURE 1:**
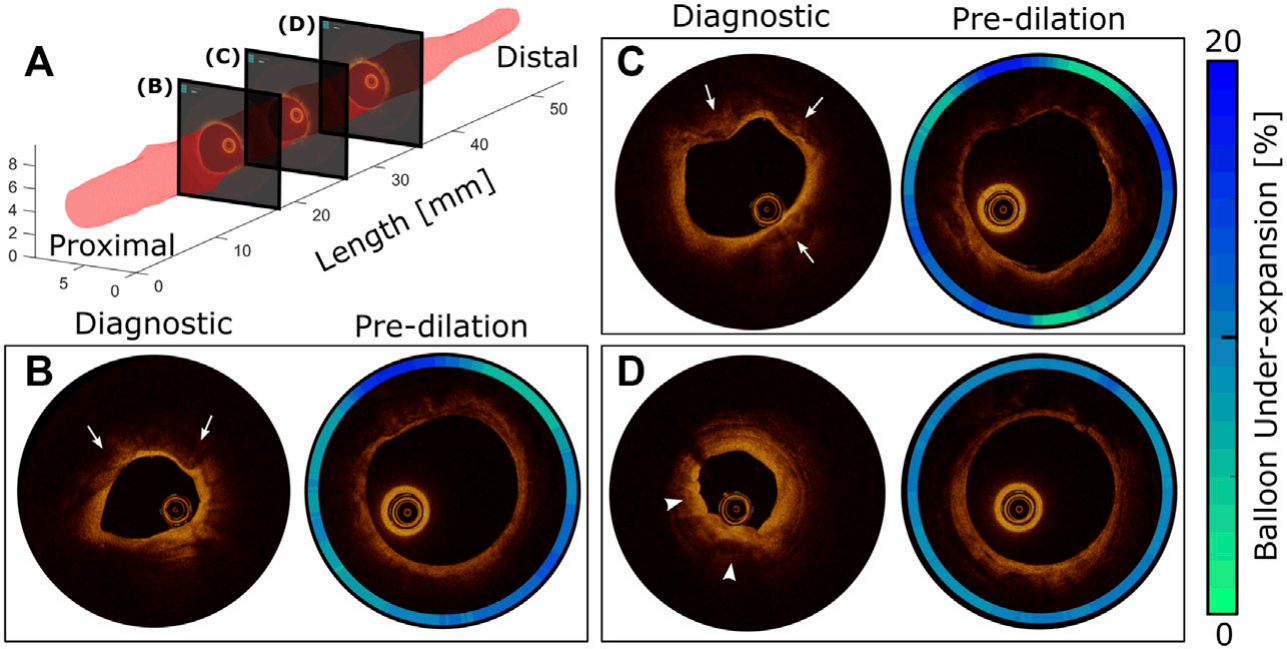
Diagnostic vs pre-dilation intravascular imaging for comprehensive morphology and material characterization of a representative target lesion. 3D representation of the artery lumen **(A)** and representative OCT slices at proximal **(B)**, middle **(C)**, and distal **(D)** locations. The presence of atherosclerosis is traditionally confirmed using diagnostic OCT conducted during diastole. According to the modified AHA criteria, the presence of fibrocalcific plaque is confirmed at slices **(B)** and **(C)** with protrusion of calcium into the lumen (arrows). Early stage fibroatheroma is also observed (arrowheads) toward the distal region **(D)** as characterized by superficial high backscattering bright signal and pronounced signal attenuation of light in the deeper plaque regions, which is probably secondary to the presence of activated macrophages, calcification, or both. After balloon inflation to 8ATM, the same artery segments were imaged, and the effect of local stiffness was quantified using balloon under-expansion relative to the diameter at 8ATM in an associated compliance table for the angioplasty balloon (contour plots).

After exempt determination by the Institutional Review Board of Medical College of Wisconsin, epicardial coronary arteries were collected from human donor hearts received post-mortem. To maintain structural and material characteristics during the experimental protocol, samples were stored in 4°C physiological salt solution (2 g Glucose, 24 mL 0.1M MgSO_4_7H_2_O, 32 mL 0.1M CaCl_2_2H_2_O mixed in 2L of a stock solution comprised of 163.69 g NaCl, 7.01 g KCl, 3.40 g Na_2_HPO_4_, 8.37 MOPS, and 2 mL 0.2M EDTA, pH adjusted to 7.4 at 37°C using 1M NaOH). A total of 6 human donor hearts were received from which 2 right coronary arteries (RCA) and 7 left anterior descending coronary arteries (LAD) were dissected (total *n* = 9). Patient characteristics are summarized in Table 1. After careful removal of the perivascular tissue and ligation of side branches, arteries were pinned down at their *in vivo* length measured prior to dissection and submerged in physiological salt solution. Arteries were pinned down straight for ease in registration of images obtained at different balloon expansions. A 6 Fr introducer sheath was positioned at both ends and fixed via suture for advancement a 0.014 guide wire and OCT imaging wire. Arteries were perfused at the diastolic blood pressure (DBP) obtained from clinical records to represent diagnostic dimensions *in vivo*. Intravascular OCT was then performed (540 frames 0.1 mm longitudinal increment, total pullback length of 54 mm) to characterize morphology and composition of the target lesion according to the modified AHA criteria (Otsuka et al., 2014). The pullback region was noted with tissue marking dye to repeatedly image the same section and for accurate sample preparation at the locations imaged. For OCT-integrated angioplasty imaging, the introducer sheath was removed, the angioplasty balloon was advanced to the segment where OCT imaging at the DBP was initiated, and pre-dilation using an 8 ATM inflation pressure was initiated (referred to as pre-dilation hereafter). The comparison of physiological intravascular OCT at DBP perfusion relative to OCT-integrated balloon angioplasty at 8 ATM inflation, as indicated in Figure 1, allowed for identification of luminal expansion from the physiological to super-physiological range of deformations applied through PCI.

**TABLE 1:** Patient characteristics.

	Sex	Age [years]	BMI [kg/m^2^]	MLA [mm^2^]	DBP [mmHg]	SBP [mmHg]	HR [bpm]	Num. of Arteries	
								LAD	RCA
Patient 1	M	42	26.4	7.23	77	144	110	1	0
Patient 2	M	70	32	8.41	57	116	91	1	0
Patient 3	M	51	26.6	4.93	71	153	99	2	1
Patient 4	M	61	21.4	5.52	NA^a^	NA	NA	1	0
Patient 5	F	71	21.5	6.02	55	117	78	1	0
Patient 6	M	51	44.8	3.5	62	115	90	1	1
Mean		57.7	28.8	5.94	64.4	129	93.6		
±SD		11.6	8.88	1.73	9.37	18.1	11.8		

BMI: body mass index, bpm: beats per minute, DBP: diastolic blood pressure, HR: heart rate, LAD: left anterior descending, RCA: right coronary artery.

^a^ blood pressure and heart rate data not available.

### 2.3 Balloon under-expansion quantification

Balloon under-expansion at each cross-sectional OCT frame relative to the 8 ATM nominal diameter reported in an associated compliance table was quantified as a surrogate for local stiffness within each artery. To quantify balloon under-expansion, a binary luminal mask was created for each OCT frame as identified mechanistically using a fast artificial intelligence solution (apeer.com). Specifically, a U-net deep learning algorithm was trained using a random subset of OCT frames (*n* = 100) manually annotated for the lumen region and divided into 80% training and 20% testing subsets. The trained U-net model provided a final loss function value of 7 × 10^-3^, accuracy of 0.999, and intersection over union of 0.997 all indicating the high predictive performance of the trained algorithm. An in-house MATLAB script was then developed to calculate balloon under-expansion from the luminal surface identified by the trained machine learning algorithm as a function of balloon radius relative to the corresponding nominal pre-dilation diameter. Tissue samples were then dissected at regions of interest identified by OCT and prepared for experimental characterization of material properties. Any under-expansion identified locally was correlated with measured material properties from biaxial extension testing using the methods discussed in more detail below.

### 2.4 Biaxial extension testing

Atherosclerotic coronary artery sections were cut and equibiaxially loaded to determine hyperelastic behavior at strains applied during PCI (i.e., super-physiological range of deformation). Samples were carefully selected from regions where plaque was large enough reduce error from heterogeneity during empirical characterization of material properties. More specifically, square samples measuring at least 8 × 8 mm (*n* = 13) were mounted at each face onto 4.7 mm bio-rakes that were magnetically attached to a biaxial tensile tester equipped with 1.5N load cells (CellScale, BioTester) allowing for high-precision real-time force-displacement-temperature measurement on a computer-controlled set-up adapted for small biological specimens. Samples were rested submerged at 37°C physiological salt solution to simulate hydration and temperature conditions *in vivo*. Each sample was preconditioned equibiaxially at a strain rate of 1% per second to 5% strain (4 repetition, 5 cycles total) where a preload of 20 mN was set initially to ensure minimum tension and accurate measurement of stress-free dimensions. The protocol was repeated by incrementally increasing the maximum strain to 10, 15, 20, and 25% strains (4 repetition, 5 cycles total). Force/displacement measurements were recorded at 30 Hz during testing while images were recorded at 5 Hz. Loading and unloading used a ramp function with 5 s rest in between cycles.

### 2.5 Constitutive hyperelastic modeling

Samples were assumed anisotropic with mixed Neo-Hookean and Exponential behavior with strain energy density function Ψ given as (Holzapfel et al., 2005):

$$\Psi =\Psi_{neo-hookean}+\Psi_{exponential},$$

$$\Psi_{neo-hookean}=\frac{\mu }{2}\left(I_{1}-3\right),$$

$$\Psi_{exponential}=\frac{k_{1}}{k_{2}}\exp\left(\left\{k_{2}\left(\left(1-\rho \right){\left(I_{1}-3\right)}^{2}+\rho {\left(I_{4}-1\right)}^{2}\right)\right\}-1\right)$$ (1)

where μ and $k_{1}$ are the Neo-Hookean and Exponential stress-like constitutive parameters and $k_{2}$ and ρ are nondimensional parameters. $I_{1}$ and $I_{4}$ are the first and fourth invariants of the right Cauchy-Green strain tensor:

$$I_{1}=\lambda_{1}^{2}+\lambda_{2}^{2}+\lambda_{3}^{2},$$

$$I_{4}=\lambda_{1}^{2}\cos^{2}\left(\alpha \right)+\lambda_{2}^{2}\sin^{2}\left(\alpha \right)$$ (2)

While anisotropic material properties can arise from collagen fibers orientation, it is challenging to quantify the spatial heterogeneity and composition of atherosclerotic samples. Therefore, anisotropy was phenomenologically accounted for through parameter α (radian). Deformation was described in terms of the principal stretches $\lambda_{1}$ , $\lambda_{2}$ , and $\lambda_{3}$ . Assuming incompressibility ( $\lambda_{1}\lambda_{2}\lambda_{3}=1$ ) and equibiaxial extension:

$$\lambda_{1}=\lambda_{2}=\lambda ,\lambda_{3}=1/\lambda^{2}$$ (3)

Together, Eqs (1–3) allowed for determination of strain energy density from empirically measured deformations for each sample to calculate model stress, $\sigma_{model}$ :

$$\sigma_{ii,model}=\lambda_{i}\frac{\partial \Psi }{\lambda_{i}}-\lambda_{3}\frac{\partial \Psi }{\lambda_{3}},i=1,2$$ (4)

Material constants was then obtained by minimizing the objective function, ϵ :

$$\epsilon =2N\times \frac{\sqrt{\frac{\chi^{2}}{N-m}}}{\sum_{1}^{N}\left\{\sigma_{11,\exp}+\sigma_{22,\exp}\right\}},\chi^{2}=\sum_{1}^{N}\left\{{\left(\sigma_{11,model}-\sigma_{11,\exp}\right)}^{2}+{\left(\sigma_{22,model}-\sigma_{22,\exp}\right)}^{2}\right\},N=Number\;of\;data\;points$$

m=number of constitutive parameters (5)

The MATLAB genetic algorithm was used for nonlinear constrained optimization with tolerance level and convergence criteria set to 10^-8^ and central numerical differentiation step size of 10^-4^.

### 2.6 Statistical analysis

Descriptive statistics are presented for continuous variables as mean ± standard deviation and for regression coefficients as *p*-values and 95% confidence intervals (CI). Unbalanced one-way ANOVA was used to assess significant differences between groups. Pearson’s correlation and linear regression analysis examined the relationship between balloon under-expansion and constitutive hyperelastic parameters describing material properties of the coronary artery lesions.

## 3 Results

Figure 1A shows the luminal surface of an example LAD coronary artery characterized through OCT conducted at the DBP (Table 1, Patient 1). Cross-sectional views of the artery are also shown for three longitudinal locations (Figures 1B–D) obtained during using the OCT-integrated angioplasty balloon. OCT identified the presence of fibrocalcific plaque at slices B and C, while distal regions of the artery (Figure 1D) more likely represent early stage fibroatheroma. Images taken at the same locations during pre-dilation identified spatial heterogeneity and balloon under-expansion relative to the nominal diameter that are represented in contour plots of Figures 1B–D. Considering under-expansion as a surrogate for local stiffness of the lesion, spatial variations observed were interpreted as differences in material properties across the target lesion. For example, at regions of fibrocalcific plaque (Figures 1B,C), more prominent radial resistance with up to 20% balloon under-expansion was observed. On the other hand, at distal regions with early stage fibroatheroma (Figure 1D), a more uniform distribution of under-expansion up to 10% was observed indicating less radial resistance and angular heterogeneity. Overall, the luminal expansion from diagnostic to pre-dilation images (i.e., DBP to 8 ATM via balloon inflation) were 65, 27, and 85%, respectively for each location. These results suggest the overall radial stiffness is higher at the proximal to middle section of the target lesion for Patient 1, while the distal region is more elastic with the highest luminal expansion flexibility. Lesions from other patients were similarly processed.

**FIGURE 2:**
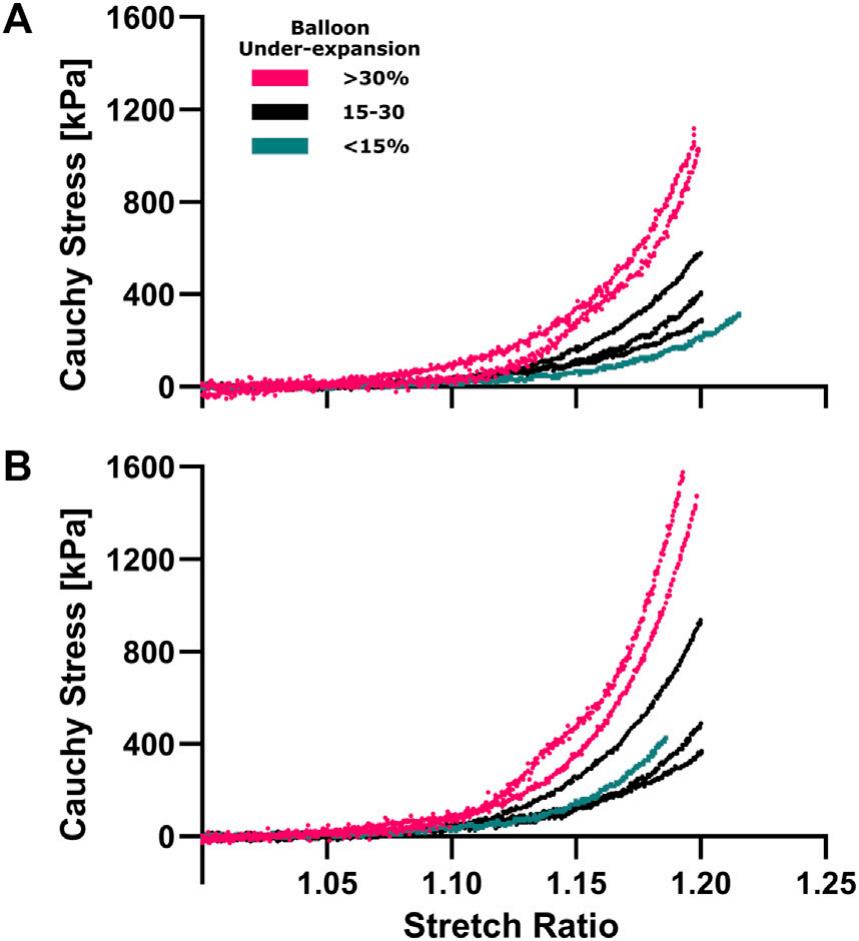
Hyperelastic characterization of coronary plaques in the circumferential **(A)** and longitudinal **(B)** directions. Equiaxial extension testing was performed to characterize material properties corresponding to under-expansion levels that are color coded for three range of <15% (aqua), 15%–30% (navy) and >30% (pink). Cauchy stress vs stretch curves were calculated from force/displacement and sample dimension data where non-linear hyperelastic behavior was observed in both directions.

Figure 2 shows hyperelastic characterization of the coronary plaques (*n* = 13) in circumferential and longitudinal directions for locations where balloon under-expansion was observed. For general comparison, Cauchy stress was compared close to the knee point, i.e., point of maximum curvature, where the transition from linear to hyperelastic portions of the stress-strain curves were observed (Claes et al., 2010), i.e., at 1.15 stretch ratio in both directions according to the current datasets. The average circumferential Cauchy stress was 66.5, 130.2, and 300.4 kPa for regions with <15, 15-30, and >30% balloon under-expansion. Similarly, the average longitudinal Cauchy stress was 68.1, 172.6, and 412.7 kPa, respectively. Consequently, strong Pearson Correlation Coefficients of 0.90 and 0.89 were observed between balloon under-expansion and stiffness for circumferential and longitudinal directions, respectively.

The circumferential/longitudinal stress ratios were 0.98 ± 0.12, 0.79 ± 0.14, 0.76 ± 0.30 for <15, 15-30, and >30% under-expansion, respectfully, indicating mild anisotropy with a general pattern of stiffness toward the longitudinal direction. Therefore, the corresponding constitutive parameter ψ was fixed at 1.22 radians to reflect the same anisotropy level while other non-dimensional constitutive parameters (i.e., $k_{2}$ and ρ ) were fixed at 8.21 ad 0.25 according to the literature (Holzapfel et al., 2005). These simplifications reduced sensitivity while tuning the Neo-Hookean and Exponential stress-like constitutive parameters of μ and $k_{1}$ listed in Table 2. Results showed significant differences between stress-like constitutive parameters obtained from regions with <15, 15-30, and above 30% under-expansion with *p*-values of 0.0015 and 0.0250 for Neo-Hookean and Exponential constitutive parameters, respectively (Table 2).

**TABLE 2:** Constitutive characterization of stress-like parameters using biaxial data from atherosclerotic coronary artery plaques (n = 13).

Samples	Plaque Type^a^	% under-expansion	Constitutive Parameters		Parameter Ranges [kPa]	
			µ [kPa]	k1 [kPa]		
I	ESFA	20	0.127	61.308		
II	FCP	24	0.923	175.33	Lower Bound	
III	LSFA	27	1.929	94.783	μ	1.27E-02
IV	FCP	12	0.013	84.26	k_1_	21.6E-02
V	LSFA	38	4.053	135.45		
VI	LSFA	41	2.741	291.19	Upper Bound	
VII	PIT	25	1.83	61.308	μ	1.27E+02
VIII	PIT	17	1.114	128.58	k_1_	21.6E+02
IX	LSFA	33	1.663	144.29		
X	PIT	14	0.147	99.398	Correlation Coefficients	
XI	FCP	31	3.11	178.1	μ	0.90
XII	CN	37	2.672	210.53	k_1_	0.89
XIII	CN	49	4.113	305.14		
		< 15	0.08±0.1	91.83±10.7		
		15-30	1.18±0.7	98.18±53.8		
		>30	3.06±0.9	210.8±72.9		
		p-value	0.0015	0.025		

^a^ Classification was performed according to the modified AHA criteria for coronary atherosclerotic plaques. CN: calcified nodule, ESFA: early stage fibroatheroma, FCP: fibrocalcific plaque, LSFA: late stage fibroatheroma, PIT: pathological intimal thickening

**FIGURE 3:**
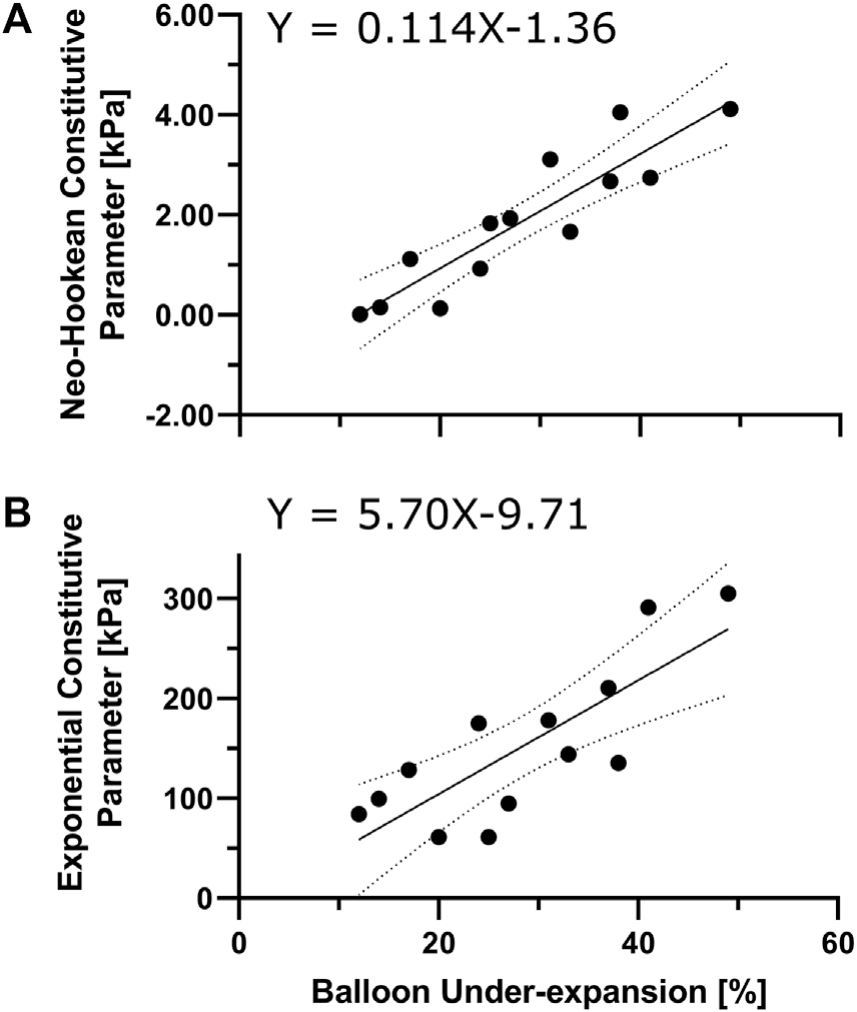
Characterization of local lesion material properties via angioplasty balloon under-expansion. Regression model to interpret neo-Hookean **(A)** and Exponential **(B)** stress-like parameters (i.e., μ and $k_{1}$ ) for direct constitutive characterization of the target lesions from angioplasty balloon under-expansion.

Figure 3 describes the transfer functions for the stiffness interpretation of balloon under-expansion at pre-dilation (i.e., 8 ATM). Under-expansion was averaged over the locations where experimental samples were dissected from with spatial resolution equal to the sample dimensions, i.e., 4.7 × 4.7 mm. Overall, a strong linear correlation was observed both in Neo-Hookean and Exponential constitutive parameters, i.e., μ and $k_{1}$ , respectively, with *R* ^2^ of 0.82 and 0.64 and significant non-zero slopes (*p*-values <0.001).

## 4 Discussion

The material properties used with computational PCI simulations often coincide with available hyperelastic constitutive relationships for a limited range of developed plaques (Kragel et al., 1989; Lee et al., 1991; Loree et al., 1994) (e.g., lipid rich, fibrous, and calcified plaque (Schroeder et al., 2004; Brodoefel et al., 2008)) as well as early stages of atherosclerosis (e.g., intimal thickening) (Holzapfel et al., 2005; Pericevic et al., 2009). The utility of this prior data is limited when the goal is, for example, to predict the likelihood of restenosis for a given patient using imaging data from the PCI period. Recent literature echoes this need for more data since arterial tissue is often obtained after autopsy when mechanical properties have degraded, and few studies account for plaque substructure in constitutive models of atherosclerotic coronary arteries (Akyildiz et al., 2014; Chen and Kassab, 2016; McKittrick et al., 2016). The current work presents a novel approach for constitutive characterization of target coronary artery lesions using a specially fabricated angioplasty balloon that allows for quantification of balloon under-expansion via OCT as a surrogate for local stiffness of the artery wall. Empirical quantification of balloon under-expansion at pre-dilation and corresponding hyperelastic material properties of the atherosclerotic plaques identified a transfer function (Figure 3) for direct constitutive characterization of coronary lesions during pre-dilation. Importantly, the inflation pressure of 8 ATM was used, consistent with the nominal pressure in many commercially available coronary artery stent delivery systems.

**FIGURE 4:**
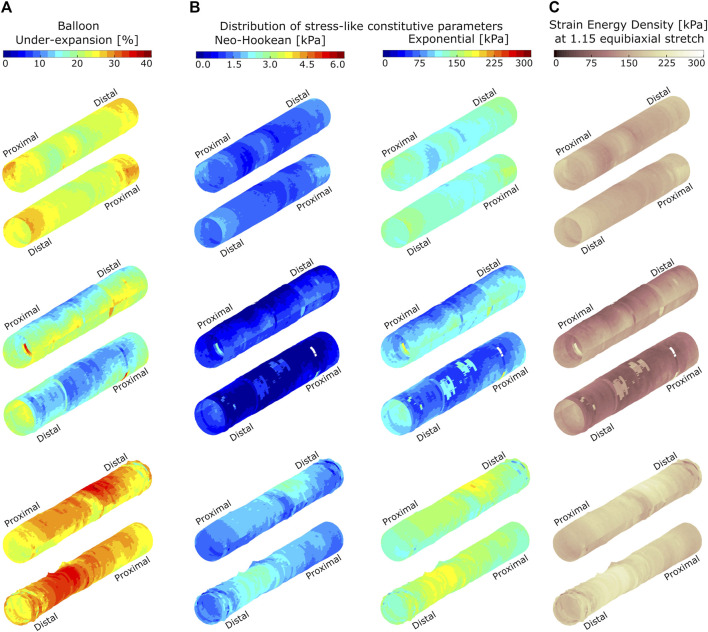
Spatial distributions of balloon under-expansion **(A)**, stress-like constitutive material parameters **(B)**, and strain energy density **(C)** for three representative atherosclerotic coronary arteries. Arteries are shown in two orientations and data are visualized on the luminal surface of the artery at pre-dilation. Balloon under-expansion was quantified as percent reduction in expanded radius relative to the expected nominal radius at 8 ATM using an associated compliance table. Stress-like Neo-Hookean and Exponential constitutive parameters are quantified through transfer functions identified empirically using biaxial extension testing results as shown in Figure 3. Strain energy density was calculated according to Eq. 1 and at a virtually applied 1.15 equibiaxial stretch ratio.

Capturing spatial variations at the micron-scale is difficult using traditional extension testing methods due to relatively large dimension of the samples (5 × 5 mm), whereas heterogeneities obtained through the proposed OCT-integrated balloon angioplasty method are at the micron-scale. Some of the image-based heterogeneity is lumped into the larger size samples dissected for experimental testing of material properties for the current work, since under-expansion was averaged over the locations where experimental samples were dissected from. Despite these differences in image-based vs. empirical resolutions, characterization of constitutive parameters resulting from Figure 3 allows for estimation of local material properties in novel ways and within a reasonable level of certainty given the infancy of the current approach. Figure 4A provides an example of the clinical utility of advancements afforded by the current work. Balloon under-expansion is shown over the luminal surface in response to pre-dilation for three representative LAD coronary arteries generally spanning the range of plaques tested for the current study. Results indicate spatial heterogeneity of balloon under-expansion that varies between arteries as a result of differences in stiffness and plaque types across patients.

Figure 4B shows the corresponding distribution of stress-like constitutive parameters (i.e., Neo-Hookean and Exponential) over the target lesions when applying the transfer functions shown in Figure 3. Figure 4C indicates the corresponding strain energy density at a virtually applied 1.15 equibiaxial stretch ratio that shows heterogenous absorption of energy when applied to the same stretch. This representation of the data can predict stent-plaque strain energy exchange that is important for accurate virtual PCI and risk assessment.

The current results from a large sample size (*n* = 13) relative prior work serve as a proof-of-concept for pre-operative patient-specific hyperelastic material characterization using a single commercially-available imaging approach (i.e., OCT) to capture hyperelastic behavior at physiologic and supra-physiologic deformations (Guvenir Torun et al., 2021; Narayanan et al., 2021) applied during PCI. The current results extend the work of Narayanan et al. who used inverse finite element methods to estimate the material properties of arterial plaque components (Narayanan et al., 2021). The authors leveraged intravascular OCT imaging data acquired during imaging wire pullback *in vivo* and an *in silico* (i.e., simulated) target geometry corresponding to 60 mmHg above the acquired geometry for three patients. Five material regions were identified based on literature including fibrous, lipid, calcium, mixed, and healthy wall tissue. The current OCT-integrated balloon angioplasty methods extend this prior work by allowing for material characterization using local balloon under-expansion as a surrogate for material properties and heterogeneity. Clinical translation of the current methods may ultimately allow for real-time material characterization that is useful clinically for several applications, such as determining when a stent with increased radial strength may need to be selected from current FDA-approved stents, or for better understanding of lesion properties in patients nearing chronic total occlusion.

The current study should be interpreted relative to several potential limitations. Difficulty in dissecting samples from morphologically distorted atherosclerotic plaques can be difficult and may result in as much as 30% loss of available samples during handling. The application of inverse computational methods such as inverse finite element analysis (iFEA) can substantially reduce lost data as well as allow for characterization of samples too small to be tested using available biaxial extension testing machines. Despite image-based under-expansion data being available at the micron-scale, these data were averaged over the dimension of prepared samples for comparison to empirical data. Hence, the spatial resolution of the current manuscript was limited to samples dimensions, i.e., 4.7 × 4.7 mm, in the reported correlation study (Figure 3). The application of iFEA in future work may allow for reducing the spatial resolution to the full potential of the proposed OCT-integrated angioplasty method, i.e., 15 microns. *In vivo* curvature was not replicated in the current methods. It is worth noting that the force exerted during balloon expansion limits curvature in the longitudinal direction, resulting in an almost straight segment. Nonetheless, future work may aim to conduct the current methods while matching *in vivo* curvature, likely using a reconstruction approach combining conventional and intravascular approaches as described previously (Slager et al., 2000; Ellwein et al., 2011; Timmins et al., 2016; Athanasiou et al., 2018; Wu et al., 2020).

## 5 Conclusion

The current advancements allow for high-fidelity constitutive characterization of coronary artery target lesions using an angioplasty balloon that accommodates the OCT imaging wire during pre-dilation. We are optimistic that state-of-the-art computational and virtual stent implantation methods can now be further advanced with patient-specific material properties identified during pre-dilation, as well as within the super-physiological range of deformations normally applied during PCI.

## Data Availability

The original contributions presented in the study are included in the article/Supplementary Material, further inquiries can be directed to the corresponding author.

## Supplementary material

Supplementary Table 1 Raw Data for Figure 2. Hyperelastic material stretch vs. stress behavior.

Supplementary Table 2 Raw data for Figure 3. Balloon under-expansion vs. stress-like constitutive hyperelastic parameters.
